# A case of peritoneal metastasis during treatment for hypopharyngeal squamous cell carcinoma

**DOI:** 10.1186/s12957-016-1025-z

**Published:** 2016-10-18

**Authors:** Takahiro Wakasaki, Hirofumi Omori, Shintaro Sueyoshi, Fumihide Rikimaru, Satoshi Toh, Kenichi Taguchi, Yuichiro Higaki, Masaru Morita, Muneyuki Masuda

**Affiliations:** 1 Department of Head and Neck Surgery, National Hospital Organization, Kyushu Cancer Center, 3-1-1 Notame, Miniami-ku, Fukuoka, 811-1395 Japan; 2 Department of Pathology, National Hospital Organization, Kyushu Cancer Center, 3-1-1 Notame, Miniami-ku, Fukuoka, 811-1395 Japan; 3 Department of Gastroenterological Surgery, National Hospital Organization, Kyushu Cancer Center, 3-1-1 Notame, Miniami-ku, Fukuoka, 811-1395 Japan

**Keywords:** Head and neck squamous cell carcinoma, Hypopharyngeal carcinoma, Acute abdomen, Peritoneal metastasis, Peritoneal carcinomatosis

## Abstract

**Background:**

Advanced head and neck squamous cell carcinomas frequently develop distant metastases to limited organs, including the lungs, bone, mediastinal lymph nodes, brain, and liver. Peritoneal carcinomatosis as an initial distant metastasis from hypopharyngeal squamous cell carcinoma is quite rare.

**Case presentation:**

A 75-year-old man diagnosed with hypopharyngeal squamous cell carcinoma and his clinical stage was determined as T2N2cM0. Notably, the right retropharyngeal lymph node surrounded more than half of the right internal carotid artery. Concomitant conformal radiation therapy was administered for the primary hypopharyngeal lesion, and the whole neck and tumor response was evaluated at this point according to our algorithm-based chemoradioselection protocol. As the tumor responses at both the primary and lymph nodes were poor, with the right retropharyngeal lymph node in particular demonstrating mild enlargement, we performed a radical surgery: pharyngolaryngectomy, bilateral neck dissection, and reconstruction of the cervical esophagus with a free jejunal flap. Then, postoperative CRT was performed. During these therapies, the patient developed a fever and mild abdominal pain, which was associated with an increased C-reactive protein level. Contrast-enhanced computed tomography from the neck to the pelvis demonstrated mild peritoneal hypertrophy and ascites with no evidence of recurrent and/or metastatic tumor formation. We initially diagnosed acute abdomen symptoms as postoperative ileus. However, cytological examination of the refractory ascites resulted in a diagnosis of peritoneal carcinomatosis. Owing to rapid disease progress, the patient died 1.5 months after abdominal symptom onset.

**Conclusions:**

The present case is the second reported case of head and neck squamous cell carcinoma with peritoneal carcinomatosis as an incipient distant metastasis. Therefore, peritoneal carcinomatosis should be considered a differential diagnosis when acute abdomen is noted during treatment for head and neck cancers.

## Background

Reported rates of distant metastases (DMs) observed in patients with head and neck squamous cell carcinoma (HNSCC) is 8.5 %. In a large-scale study of HNSCC (*n* = 2550), Spector et al. found DMs in 16.3 % of patients with hypopharyngeal carcinoma [1]. Because nearly 100 % of DMs from HNSCCs arise in relatively limited organs: the lungs (77 %), bone (19 %), mediastinal/other lymph nodes (4 %), as well as the brain, liver, and skin, it is quite rare to encounter DMs at other sites [1]. We recently encountered a case of hypopharyngeal carcinoma that showed peritoneal metastasis (PM) as an initial DM. Based on an English language medical literature PubMed search, this appears to be the second reported case of such a condition. Herein, we present and discuss the clinical course of the patient with a review of medical references and medical charts of our institute.

## Case presentation

In 2014, a 75-year-old man was referred to our department with bilateral swelling of the cervical lymph nodes. There was moderately differentiated SCC in the posterior wall of the hypopharynx. After an extent-of-disease workup, his clinical stage was determined as T2N2cM0. Notably, the right retropharyngeal lymph node surrounded more than half of the right internal carotid artery. Initially, 41.4 Gy of concomitant conformal radiation therapy (CRT; 1.8 Gy/fr, 5 fr/week + cisplatin 80 mg/m^2^, on day 1) was administered for the primary hypopharyngeal lesion, and the whole neck and tumor response was evaluated at this point according to our algorithm-based chemoradioselection protocol [2].

As the tumor responses at both the primary and lymph nodes were poor, with the right retropharyngeal lymph node in particular demonstrating mild enlargement, we performed a radical surgery: pharyngolaryngectomy, bilateral neck dissection (rt.: Ib-V, lt.: Ib-V), and reconstruction of the cervical esophagus with a free jejunal flap. Owing to the severe adhesion of the lymph nodes, the left internal jugular vein and the left spinal accessory nerve were sacrificed.

**Fig. 1:**
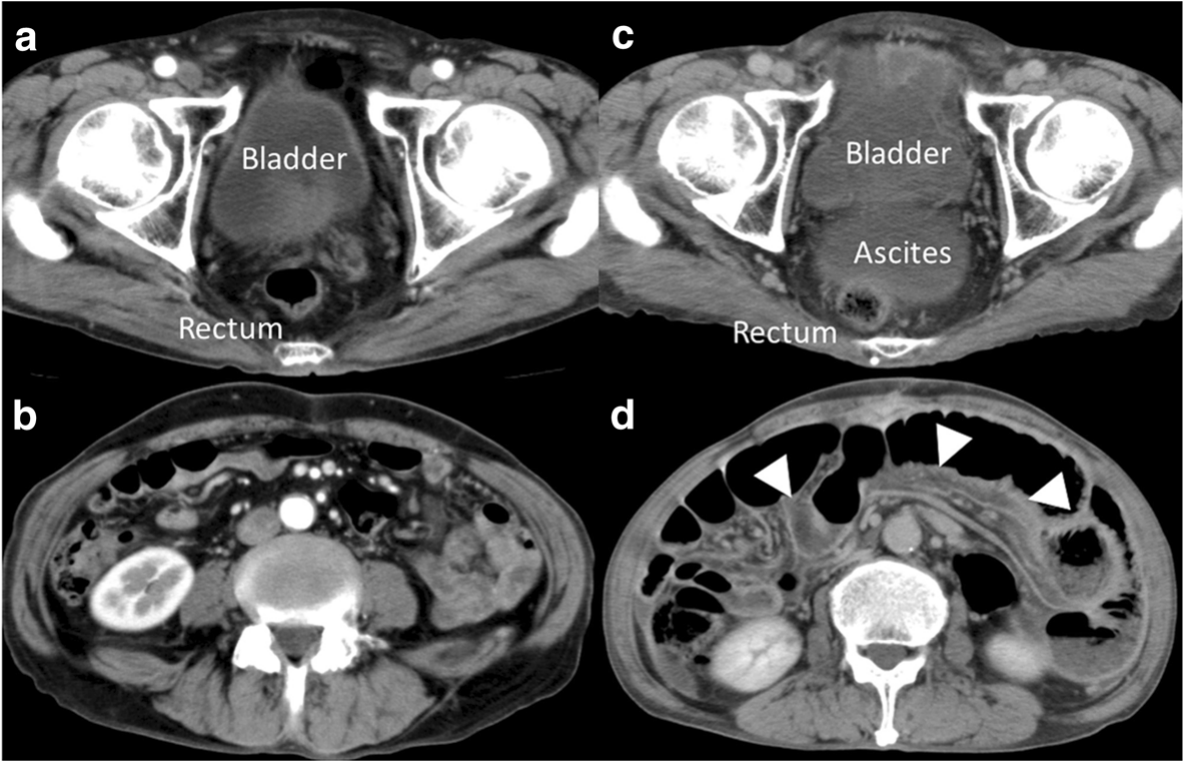
Computed tomography imaging showed the influence of peritoneal carcinomatosis. **a** The abdominal CT image before the abdominal surgery represents no findings of ascites. **b** The abdominal CT image before the abdominal surgery represents no findings of peritonitis. **c** Rectovesical excavation. **d** *Arrow heads* indicate hypertrophy and increasing fat brightness of the peritoneum. *CT* computed tomography

Pathological examination revealed pT2pN2c with extra-capsular spread of the lymph nodes. Postoperative CRT was started on postoperative day 21. However, during CRT, cystic lesions appeared around the anterior edge of the left lower trapezius muscle (i.e., posterolateral area of the dissected level V), and fine-needle aspiration cytology revealed a class IV finding. We then surgically removed the tumor, which was a recurrent lymph node. On day 10 following reoperation, the patient developed a fever and mild abdominal pain, which was associated with an increased C-reactive protein level. Contrast-enhanced computed tomography from the neck to the pelvis demonstrated mild peritoneal hypertrophy and ascites compared to the preoperative images (Fig. 1a–d) with no evidence of recurrent and/or metastatic tumor formation.

**Fig. 2:**
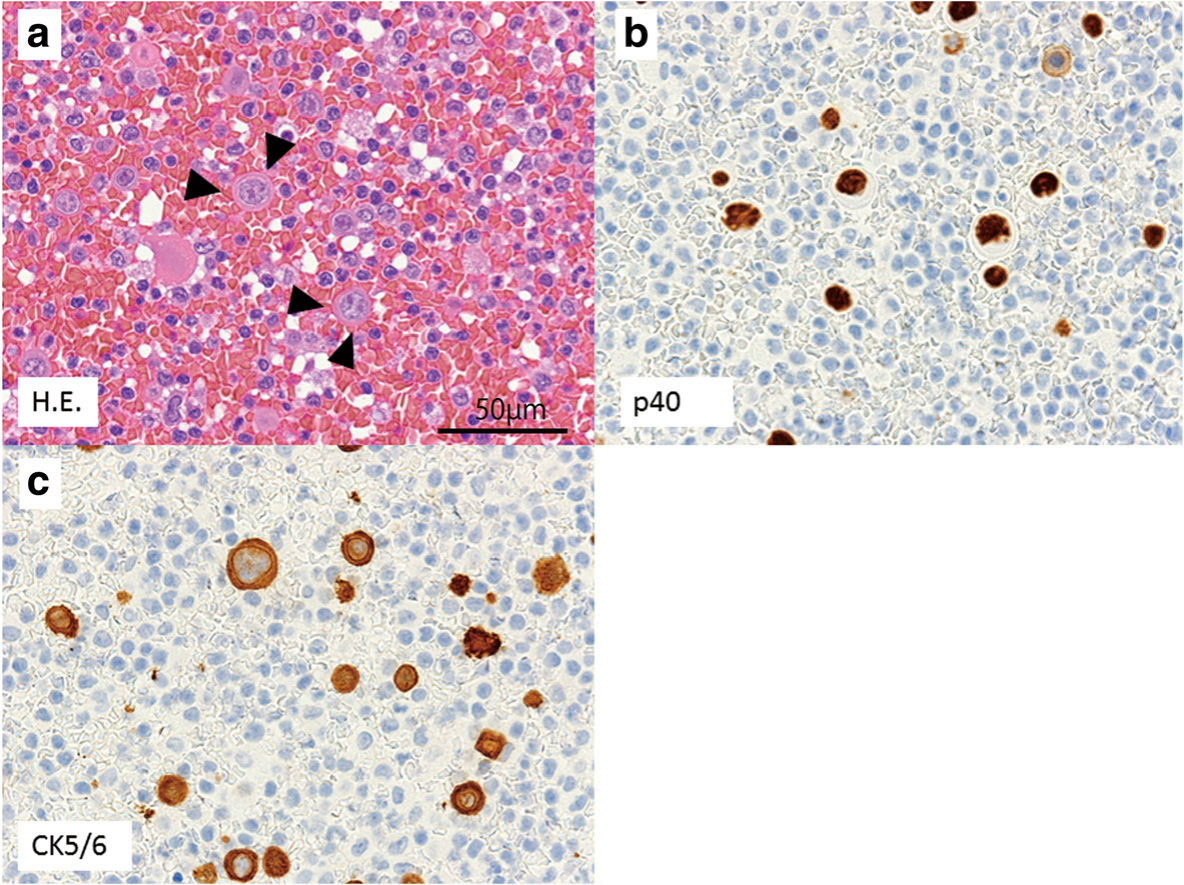
Cytological microscopic imaging of peritoneal fluid (cell blocks). **a** Hematoxylin-eosin stain showed isolated atypical cells with nucleoli distinct within the nucleus (*arrowhead*). **b** Immunohistochemistry for p40, a highly specific squamous cell carcinoma marker, was positive in the nucleus. **c** Immunohistochemistry for CK5/6, epithelial markers, was positive in the cytoplasm. *H.E.* hematoxylin-eosin

Assuming postoperative ileus, the patient was asked to fast and was treated with an antibiotic. However, no improvement in laboratory, imaging, or clinical findings was obtained. On day 20 after reoperation, the result of an ascites test was suggestive of SCC. For a more accurate diagnosis, cellblocks were made using recollected peritoneal fluid. Cytological examination revealed that tumor cells were immunopositive for p40 (a highly specific squamous cell carcinoma marker) and CK5/6 (epithelial markers; Fig. 2a–c) [3]. These findings, together with his clinical course, indicated the development of PM from hypopharyngeal squamous cell carcinoma. Considering the patient’s rapidly deteriorating general condition, the best supportive care was chosen. However, the patient died 1.5 months following the onset of peritonitis symptoms.

### Discussion

PM often results from cancers of peritoneal organs, including gastric cancer, colon cancer, pancreatic/biliary cancer, ovarian cancer, and uterine cancer [4]. As a result, almost all cases of PMs are adenocarcinomas. Only a few cases of SCC-origin PMs, derived from the esophagus, uterus, and ovaries, have been reported [5–7].

We reviewed medical charts of HNSCC patients treated at our institute from January 1995 until June 2015 and found that 258 patients developed DMs. Other than the present case, only one additional patient manifested PM. In the prior case, PM occurred 2 years after initial treatment in association with concomitant pulmonary, bone, and skin metastasis. In contrast, the present case developed PM as an initial DM from hypopharyngeal squamous cell carcinoma.

A PubMed search revealed three reported cases of PM from HNSCC [8, 9]. Wong et al. reported a case in which PM occurred following chemoradiotherapy for hypopharyngeal carcinoma [8]. In addition, Hsu et al. reported two cases, both of which were associated with multiple DMs to other organs [9]. Thus, the present study appears to be the fourth reported case of PM from HNSCC and the second reported case of PM occurring as an initial metastasis from HNSCC.

In the previously reported cases, after the onset of peritoneal carcinomatosis, the prognoses of the patients were very poor, ranging from a few days to a few months [8, 9]. In the present case, the patient also demonstrated unfavorable progress and died 1.5 months following the onset of acute abdomen. In general, malignant ascites and PM progress rapidly. However, in selected cases of gastrointestinal and gynecologic adenocarcinomas, chemotherapy and peritoneal excision can improve quality of life and provide a survival benefit [10, 11]. In addition, cell-free and concentrated ascites reinfusion therapy is efficacious for treating intractable ascites, because it relieves abdominal symptoms and maintains plasma osmolality, thus sparing the frequent removal of large volumes of peritoneal fluid [12]. However, because of the rapid disease progression in the present case, these options were not feasible. In any case, the establishment of effective treatment modalities for patients with PM from HNSCC, which progresses extremely rapidly, is an urgent task.

We initially misdiagnosed the patient’s condition as postoperative ileus, mainly because it is not uncommon for patients to develop ileus following the harvest of a free jejunal flap and because there was no evidence of tumor formation in the peritoneum or pelvis. However, the cell block-based peritoneal fluid cytology test led to the diagnosis of PM, sparing invasive procedures (e.g., laparotomy). Therefore, when encountering a refractory acute abdomen during and after treatment for HNSCC, it is essential to consider the possibility of PM and conduct an ascites test, despite no apparent tumor in the abdominal cavity.

Given that the present case underwent cervical and abdominal surgery simultaneously, it is possible that iatrogenic plantation of cancer might be the cause of PM. However, in our institute, the surgical field and instruments are strictly separated to avoid cross-contamination. This principle has worked well thus far, and we have never encountered any cross-contamination-related condition considering our 79 cases of free jejunal flap reconstruction performed during the last decade. Therefore, we assume that iatrogenic transplantation is not the cause of PM in the present case.

Reported risk factors for distant metastasis of HNSCC include extracapsular invasion in the neck, advanced lymph node metastasis (N2, N3), a long period of lymph node metastasis, spread of lymph node metastasis to the lower neck, advanced primary lesion, and absence of local control [13]. Notably, the present case met all these criteria, suggesting a high metastatic potential of the tumor cells. This may partly explain the unusual metastatic pattern seen in the present case.

## Conclusions

We reported herein an extremely rare case of hypopharyngeal carcinoma, which demonstrated PM as an incipient metastasis during curative treatment. Although rare, PM should be considered when ascites or peritonitis occurs during or after treatment for HNSCC.

## Abbreviations

DMs: Distant metastases
HNSCC: Head and neck squamous cell carcinoma
PM: Peritoneal metastasis
